# Migration and Health in the Construction Industry: Culturally Centering Voices of Bangladeshi Workers in Singapore

**DOI:** 10.3390/ijerph14020132

**Published:** 2017-01-29

**Authors:** Mohan J. Dutta

**Affiliations:** Center for Culture-Centered Approach to Research and Evaluation, Department of Communications & New Media, National University of Singapore, Singapore 117416, Singapore; cnmmohan@nus.edu.sg

**Keywords:** workplace risks, injury, workplace safety, culture-centered approach, migration, health

## Abstract

Construction workers globally face disproportionate threats to health and wellbeing, constituted by the nature of the work they perform. The workplace fatalities and lost-time injuries experienced by construction workers are significantly greater than in other forms of work. This paper draws on the culture-centered approach (CCA) to dialogically articulate meanings of workplace risks and injuries, voiced by Bangladeshi migrant construction workers in Singapore. The narratives voiced by the participants suggest an ecological approach to workplace injuries in the construction industries, attending to food insecurity, lack of sleep, transportation, etc. as contextual features of work that shape the risks experienced at work. Moreover, participant voices point to the barriers in communication, lack of understanding, and experiences of incivility as features of work that constitute the ways in which they experience injury risks. The overarching discourses of productivity and efficiency constitute a broader climate of threats to worker safety and health.

## 1. Introduction

Construction workers globally face disproportionate threats to health and wellbeing, constituted by the nature of the work they perform [1]. The workplace fatalities and lost-time injuries experienced by construction workers are significantly greater than in other forms of work [2]. The productivity-driven and efficiency-based nature of the construction industries place tremendous burden on the health of workers [2,3]. Add to this the large number of migrants that work in the construction industries globally, contributing further to the precarious nature of construction work, marked by limited protections and the lack of access to resources (such as health care, insurance etc.) that are otherwise guaranteed to citizens [4,5]. Essential to cotemporary global patterns of migration and construction work are the exploitative conditions of global labour in the construction industries, often taking advantage of flexible labour-related policies that offer little protection to migrants [6,7]. The low and semi-skilled nature of migrant construction work is accompanied then by the contractual nature of the work in global economic flows, and the productivity-driven pressures that are placed on construction workers.

In the dominant health communication literature on workplace safety and health, the normative idea of workplace safety focuses on promoting individual behavior change in workers through the development of effective messages [8,9]. Salient in this framework is the conceptualization of communication as promotion of individual behavior change, placing emphasis on the safety training of workers [10,11]. Workers are targeted through informative and/or persuasive messages, with the emphasis of health communication therefore in developing effective messaging strategies [12,13,14,15]. Safety is conceptualized in terms of specific behaviors that are promoted in the target population of workers, tied to awareness [12,13]. Similarly, studies on workplace safety cultures examine the perceptions held by workers regarding the support in the organizational culture for safety practices, operationalizing cultures of safety as aggregates of individualized perceptions. Missing from this framework of individual behavior change are structures of construction work and the nature of workplaces. The risks situated amidst these structures mostly remain unaccounted for in the dominant body of scholarship on workplace safety and health, with its narrow emphasis on behavior change [15,16].

On a similar note, health communication interventions targeting migrants often take migrant culture as deficit, to be targeted through effective health promotion messages [15,16,17,18]. In this framework, culture is conceptualized as a static collection of beliefs and values, mapping immigrant culture in relationship to the host culture, and developing recipes for cultural transformation through communication as persuasion [19,20,21]. Communication, connected to culture, is culturally tailored in order to adapt to the characteristics of the immigrant community, with the goal of generating culturally sensitive messages that are persuasively effective with the targeted immigrant community. Culturally sensitive messages are based on identifying characteristics of the culture that can be spoken to in order to generate affiliation and persuasive message processing. Missing often from this dominant thread of health communication are the local cultural contexts of immigrant communities [21,22,23]. In configuring immigrants as passive recipients of health messaging, the agentic capacities of immigrants are erased. Also absent from dominant health communication theorizing are the voices of local communities of immigrants, their lived experiences, and their negotiations of health. Moreover, the individualistic health communication frameworks often fail to take into account the broader political economy of immigrant life [20,24,25].

Migrant workers from Bangladesh working in the construction industry in Singapore form a part of this global flow of labour, negotiating their health amid global structures of flows of capital and labour [25,26,27]. In this manuscript, I apply the theoretical framework of the culture-centered approach (CCA) to listen to the voices of migrant construction workers in Singapore, focusing on the articulations of health, risk, and safety in the realms of construction work [20,23,25]. The concepts of culture, structure, and agency are offered as conceptual anchors for foregrounding local meanings of risk and safety in construction work. The voices of the workers presented in this manuscript attend to the structures that constitute everyday experiences of construction work, the threats to health and safety as understood by construction workers, and the potential solutions to risks at workplace that are suggested by construction workers. Rather than suggesting behavior change in the form of individual-level solutions that are typically recommended in workplace safety communication interventions, the voices of the participants articulate anchors for structural transformations that attend to the contextual features of construction work.

### 1.1. Workplace Safety, Health and Communication

The dominant approach to workplace safety in the health communication literature conceptualizes health as individualized responsibility, developing workplace safety programs and training interventions directed at promoting safety practices [8,10,20]. The overarching theoretical framework informing workplace safety interventions is driven by the impetus to create effective health promotion messages that would promote healthy behaviors in workplaces. The emphasis is placed on deciphering the appropriate communication strategies that would effectively persuade the audience of workers to practice safe workplace practices. In this backdrop of the dominant framework of workplace safety communication, the culture-centered approach (CCA) offers an entry point for exploring the ways in which local contexts shape the everyday experiences of risks among construction workers, and the lived experiences of workers with these risks [22,23].

### 1.2. Culture-Centered Approach

Health is constituted in the everyday meanings individuals and communities make of health, embedded within local contexts and in the cultural formations of community life [15,16,17]. Conceptualizing culture as dynamic, fragmented, and ever-transforming, the CCA foregrounds meanings as entry points for the constructions of health and wellbeing [15,24,25,26]. The CCA proposes that cultural contexts are anchors for understanding how health risks are understood, how they are made sense of, and the ways in which they are negotiated by community members [27,28,29,30]. Based on a dialogic approach to culture and community life, it suggests that the theorizing of health needs to be embedded within sense-making processes of local communities, developing entry points for solutions to health risks that are embedded in the everyday negotiations of structures, resources, and contexts [17].

Meanings of health and health risks are located at the intersections of culture, structure, and agency [27]. Culture is a complex web of shared practices, beliefs, and values that offers the anchor for interpretive frames. Meanings draw upon cultural resources and in turn, shape the ways in which cultural scripts are constructed and reproduced. Structure refers to the material realities that constrain and enable human action, depicting the distribution of resources within social systems [16,17]. Agency reflects the capacity of community members to make sense of structures and to negotiate these structures, drawing on cultural scripts and interpretive frames that are meaningful to them. The intersections of culture, structure, and agency depict the constraints and challenges to health experienced by community members, their sense-making processes in understanding these structures, and their day-to-day practices of negotiating these structures. This article draws from the CCA and co-constructs [25,26] meanings of health with Bangladeshi construction workers, attending to the ways in which these workers make sense of these risks in their everyday lives. Singapore has grown increasing reliant on immigrant labor in the last 15 years, with the number of foreign workers almost doubling between 2000 and 2010 [31,32]. It houses 319,000 laborers working in the construction sector alone in December 2013 [33]. The Singapore government is a major consumer of construction services through public housing development—over 17,000 flats were built in 2011 alone (SDS, 2012). Foreign workers are hired under a Work Permit (WP), which is granted to foreigners who wish to work in Singapore. They must also come from approved countries of origin, which are grouped into 4 categories: (1) *Malaysia*; (2) *North Asian Sources (NAS)*, which comprise Hong Kong, Macau, South Korea, and Taiwan; (3) *Non-Traditional Sources (NTS)*, which comprise India, Sri Lanka, Thailand, Bangladesh, Myanmar, and Philippines; and (4) *People’s Republic of China* (Singapore Ministry of Manpower (MOM), 2012). Employers in Singapore are required by law to meet several conditions in hiring foreign workers. One of the conditions is that employers must foot a monthly levy per unskilled worker. With regard to the health and wellbeing of the migrant workers, the Employment of Foreign Manpower Act states that employers must fulfill requisites such as ensuring healthy and safe working conditions, paying for initial medical examinations, bearing the cost of medical treatments when the worker falls sick or is injured, and purchasing and maintaining individual medical insurance (MOM, 2011). The Act also clearly disallows employers from passing on health care costs and other employer financial obligations on to the foreign employees.

## 2. Materials and Methods

The article draws on 60 ethnographic in-depth interviews (out of a total of 180 interviews, only selecting those in-depth interviews that voiced risks of injury at construction work) conducted with Bangladeshi construction workers in Singapore, part of a broader ongoing culture-centered ethnographic project that was initiated in 2008 and that resulted in an advocacy campaign on worker rights in 2014–2015. The construction workers who participated in the project were selected through the snowball sampling strategy and reflect a variety of subsectors (e.g., residential construction, commercial, public works), and trades (e.g., masonry, ironwork, electrician). The project was approved by the University’s Institutional Review Board (IRB) (NUS-IRB Ref 12-392) and informed consent was secured from the participants. The in-depth interviews ranged from 40 min to two hours in length, with an average interview being 55 min in length. The interviews were conducted in Bengali and then translated into English. The in-depth interviews that this paper draws on resulted in 725 pages of single-spaced transcripts. In addition to the in-depth interviews, I conducted participant observations at public spaces where Bangladeshi construction workers hang out, spending time in Little India (space in Singapore where migrant workers gather, especially on their day off on Sundays), attending performances put together by migrant workers, accompanying migrant workers during their visits to run errands, accompanying migrant workers during visits to doctors, and coordinating broader discussions with migrant workers as part of the larger culture-centered project driven toward identifying problems and developing solutions. The narratives shared by the participants in the project point toward the structural contexts of migrant construction work, and the threats to health and safety that are constituted amid these structures. In this article, I will share snippets from these conversations to elucidate the interplays of structure and agency in articulations of migrant worker health, wellbeing and safety within the broader ambits of construction work.

## 3. Results

The narratives shared by the participants point toward their everyday struggles for negotiating health amid the structures of migrant construction work. The everyday meanings of health are situated amid the local cultural understandings of food, culturally embedded meanings of and approaches to health, and the structures of construction work that shape the distribution of resources. In sharing their experiences with structures, participants point toward the profit-driven framework of the structures that often directly threaten human health and wellbeing. Moreover, participants share their strategies of everyday enactments of agency.

### 3.1. Health and Workplace Injuries

Health is constituted in the ambits of the everyday risks of doing construction work. For many workers, workplace injuries are salient in their lives in the construction industry. *Abdul* (the names of the participants have been changed to protect their identity) shares, “These injuries are part of our daily life. That you will get hurt while working is something you don’t worry about. You know that these things happen.” Similarly, shares *Jamal*, “I had an accident while lifting a bag of cement. I was trying to hurry. I know this happens to you if you are working in this industry.” The participants point toward the notion of risks to safety as embedded in their day-to-day work, attaching meaning to risk at work in the ambits of the structures of work in the construction industry. As shared by *Kaseem*, “This work, I work on tall buildings, there is risk every day. When I am working at height, there is always the chance of fall. You don’t think about that.” For *Kaseem*, knowing that his work is by nature risky, constitutes his “not thinking about work.” That risk is an everyday part of their lives as workers in the construction industries shapes the everyday negotiations of risk. He further notes, “It’s the wish of *Allah*.” This culturally-embedded religious narrative of workplace risk is situated in relationship to the structures of work on tall buildings. The structure of construction work constitutes the sense of inevitability that is articulated in a narrative of religiosity. Similarly, *Bashir* shares:

I know that I have to do this kind of work. It is tough. I know it is difficult and there are risks in the different things I do. If I just worried about how difficult the work is, or what the risks are, I would not be able to do the work. I would be scared, and try to avoid it. But my family needs the money. So I have to do this work. I am making the money and sending home every month. I don’t really think about anything else.

This notion of not really thinking about the risks of work is shared by a number of participants. Note in these narratives shared by the participants the ways in which they understand risks to health and safety as part of their everyday lives, pointing to the overarching structural context of deprivation and familial needs within which they voice not really thinking about the risks of work. The cultural articulation of familial ties and family needs as the reason for work shapes the meanings of inevitability the workers construct around workplace risks. For *Abdul*, “I have come here to earn and send money home. I took a big loan, my wife put all her jewellery for mortgage to get loan. So now I just have to do the work. If it is risky, I can’t worry about it.” *Abdul’s* sense of not worrying about the risk ties the structure of construction work with his cultural values of earning money to care for family and send money home.

At the same time, participants share that workplace injuries are life events that can transform their lives and push them into immobility. They share stories of co-workers who have been killed in workplace accidents. *Maqbool* witnessed a co-worker killed in an accident, and shared the following: “It just happened. I was going with my work. And then this sound. He had fallen. When things like this happen, they change everything. I know this can happen to me.” Similarly, for *Bashir*, the death of a co-worker drives home the vulnerable conditions of construction work, “I know that this can happen to me as well.” This is also shared by *Arnab*, “I have seen a number of these accidents take place in my worksite. Some are life threatening. One time, *Robin*, he was working right in the next station, and then some friends came and said, he has been crushed by the cement mixer. I ran. He was dead by then.” Sharing similar experience of having witnessed a co-worker lose a toe, *Bimal* notes, “When you are working in construction, these things can happen. You just know that they can happen.” Note here the paradox between the articulation of “not thinking about the risks to health” and “being aware of the vulnerabilities to health constituted amid everyday work.” Participants on one hand suggest that they don’t really think about workplace injuries, and on the other hand, are well aware of the everydayness of injuries as routinized into their jobs. In participants’ stories of workplace deaths and workplace injury-related disabilities, the costs of injuries to them and to their families in Bangladesh emerge strongly. Participants note how injuries at work are significant threats to their health, livelihoods, and economic mobilities of their families.

### 3.2. Workplace Injuries and Health Seeking

For a number of participants in this ethnographic project, workplace injuries present challenges in the realm of seeking treatment, after care, and securing adequate medical leave. Shares *Sobuj*, “When I dropped a plank on my feet, it hurt a lot. The supervisor gave some first aid and then said it will be alright. Then that night, it swelled. I did not know where to go for treatment. The supervisor said it will become better, just give it some time.” *Sobuj* did not have access to medical treatment and did not know how to secure access to a health provider. For a number of participants, the supervisor ignored the injury and asked the worker to get back to work. Also participants noted the ways in which the limited access to health information (about health resources and where to locate them) shaped their inability to seek out treatment after an injury.

*Ahmed* voiced the following narrative, “I broke my toe one day while carrying a load and fell down. I had to be in rest, and could not go to work. Did not get my salary for those days.” *Ahmed’s* experience of workplace injuries is intertwined with experiences of economic vulnerabilities at work shared in the previous section. Having a workplace injury meant the inability to earn money, which translated into a weakened ability to send money back home to family. He went on to note, “This month, I will have very little to send home. So no matter what, I just let it get better a little and went back [to work].” Even though Ahmed was still experiencing the pain, he noted that being absent from work any longer would mean his family in Bangladesh wouldn’t have the resources needed for the coming month. The physical threats to health posed by workplace injuries translate into worries about what would happen to their economic condition and to their ability to return the debt or take care of their families. This means that in spite of their injuries, workers often go back to work just to earn the money needed to support their families. The culturally-situated articulations of familial responsibility shape the ways in which the workers experience workplace injuries.

Although existing policy frameworks dictate that workers qualify for medical leaves when injured, a number of participants discussed the ways in which the doctors the company arranged them to see were reluctant to issue medical certificate as that would mean less money for the company. A number of participants felt that company-assigned doctors often worked in collusion with the company. When asked why they didn’t seek other options, they often noted that they were constrained by their company with respect to which doctor they could see, and that they did not have access to adequate information about health resources that would be available to them. In other words, not being aware of the healthcare infrastructure in Singapore, they often did not know where to go to or how to navigate the structure. This point is shared by *Najmool*, “I cut my finger, and went to the company doctor because that’s where the boss [boss is a term used by migrant construction workers to refer to individuals in power at the workplace (e.g., crew leader, supervisor, owner of company, contractor)] sent me. The doctor was no good, just gave me a bandage and no MC [referring to medical certificate]. I did not know anyone else. I went back to work the next day, and my finger was still hurting.”

### 3.3. Efficiency and Workplace Risk

The experiences of workplace injuries are constituted in the backdrop of the risks that are built into workplaces in Singapore. Participants share that the risks to their everyday health and safety are often posed by the productivity and efficiency demands experienced in the construction industry. *Sajal* shares, “The nature of construction work itself is risky. Then think about all the time pressure that shapes the work we do. There are volumes that need to be met. Certain number of things that we need to finish by a date. There are project deadlines.” The notions of project volume and project deadline emerge consistently in the interviews. For *Nabin*, “Everyone is working hard, and the boss is always pushing. The boss needs to deliver a set of complete jobs by this date. Then the boss pushes everyone to meet that target. So there is that pressure to get the work done.” Similarly, notes *Babulal*, who has worked as a supervisor, “Things need to be done efficiently. That means get as much out with as little number of workers and as little time. So there is always a push for the numbers.” This pressure of getting the job done efficiently translates into pressures on the job, with workers “rushing through tasks.” Notes *Babu*:

There are many things that need to be finished in a day. So the supervisor is always trying to meet the deadlines. Yelling at the workers to get moving quickly. To get all the things done. And there is no time. So it is easy to miss something. Or to have an accident. Because I am thinking, I need to get this, this, and this done. I have a lot of tension on my mind. I am trying to get all the things done. And that’s when I make a mistake.

The point shared by *Babu* suggests that taking care of the assigned tasks in the limited timeframe increases the risks of injury. Worth noting in the narrative is the voicing of risk tied to the errors made amidst the time and productivity pressures at work.

Further elaborating on the discourses of productivity, participants note the ways in which the overarching discourse governing their work is of performance, meeting the targets that have been established. Notes *Rasul*, “These targets are very hard to meet. They are set very high. So you are always pushing, pushing, pushing just to meet the target. Then all you are thinking about is the target.” The articulation of targets at high levels that are typically unachievable means that workers focus entirely on meeting these targets. Given the precarious nature of construction work and the uncertainties tied to work, the pressures of performance translate into the risks workers take in getting the job done in a timely fashion, meeting the pre-established deadlines.

### 3.4. Food Insecurity, Fatigue, and Workplace Safety

Moreover, participants noted their everyday lives as risks to health and threats to workplace safety. For example, for *Nabin*, to be able to carry out the heavy load of construction work, “the basic food is a must.” He shared how construction work is heavy work, and is demanding on the body, and therefore requires the adequate supply of nourishment to the body. To do such work as he did at the construction sites, often lifting heavy weight and carrying the weight for distances, called for him to have enough physical strength, which in turn, he derived from eating adequate food. Yet he noted, “Food is something that is often absent from the lives of Bangladeshi workers. I work long hours, and by the time I get back, the food that is catered is already stale. There are many nights when I don’t get to eat anything.” The participants discussed the heavy work at construction sites, juxtaposed in the backdrop of the absence of sufficient food from their everyday lives. This for many participants led to injuries in workplaces, producing fatigue on the job. This point was elucidated by *Jahan*, “Without the food, there is no energy left to do the work. I feel tired. And because I am tired, I am going to make some error on the job.” For *Mohsin*, “without the food, also because I skip a meal when the food has gone stale, it affects my health.” On a similar note, *Kareem* shares, “The hard work in the construction site goes from 7 in the morning until 8 or 9 in the evening. This is hard work. How can I do the work when I don’t have enough food in my body and feel lethargic? I feel very tired and then worry that I am going to fall or have an accident. My stomach is empty.” The hard work at construction sites is juxtaposed in the backdrop of the absence of quality food, which in turn results in the feeling of lethargy and the increased likelihood of making errors on the job.

Bangladeshi construction workers in Singapore often live in dormitories, and this shapes the overarching context of inaccess to food. While there are a wide range of dormitories in Singapore, in most of these dormitories, there are no cooking facilities. As a result, most of the workers receive food from catering agencies. There are a range of catering agencies that operate in Singapore, and the extent of regulation of catering agencies varies. Whereas some of the catering agencies are licensed, there are also a number of catering agencies that are not licensed. Workers typically learn about and sign up for catering agencies through middle men, who can be other more senior workers, security and other personnel at the dormitory, or colleagues through workplaces. *Rezan* shares his dependence on the catering companies, “We can’t cook our own food in the dormitory. There is no facility to cook own food. So I will have to depend on the catering company. Even though the quality of the food delivered by the catering company is of poor quality, I have no other choice.” Many Bangladeshi construction workers share similar problems with the quality of the food delivered by the catering company. This is what *Zoeb* shares, “The problem is with the workers having to get the food from a catering company, and the catering company does not really care about the quality of the food we get. It just gets something to the workers. For most days, I can’t just eat the food because it has gone stale.” Workers share that the lunch, which they usually eat at work around noon, is usually delivered early in the morning before they take the transport to work, and is cooked the night before. As a result, the cooked food often goes stale. Shares *Bilkis*, “I end up not eating the food many times, just skipping it because it has gone stale.”

Participants share stories of stale or unhygienic food that results in sickness. Shares *Gholam*:

The food is so unhealthy that it can get you sick. There are many times I have had to take a medical certificate (MC) for a leave because I have gotten sick from eating the food. One time, it was stale. I knew it when I ate it. But I was so hungry that I ate it. Later it made me sick.” In the narrative shared by *Mustaq*, we hear the experience of unhealthy food that made him sick. Along similar lines, *Saleem* shares, “The food has often gone stale by the time I eat it. It is delivered in the dorm at 5 a.m. in the morning and is cooked the previous night. I eat it only at noon during lunch time and that’s when it has already become stale. So many times I just skipped the meal because the food had gone bad.

The story of food that has gone bad is shared by a number of participants. The poor quality of food emerges as a consistent theme in participant narratives of health, situating food within their cultural contexts and depicting the structures of the dormitories and catering companies that limit their access to healthy food.

The sense of food insecurity then is tied to articulations of fatigue. Notes *Mahmood*:

I have not eaten proper food for many days. You look at this food. It has gone bad. How can you expect workers to eat this food and still carry on with work? I am very tired most of the time. I just don’t have the energy, or I am feeling sick from the bad food. This makes me make mistakes at work.

The storying of lack of access to quality food is tied to experiences of poor health and fatigue shared by workers. Moreover, experiences of poor health and fatigue are tied to risks of workplace injuries. The centrality of food in the narratives voiced by the participants reflects the broader cultural constructions of food in Bangladesh. Moreover, given the importance placed on food, having access to culturally appropriate food emerges as a risk for workplace injuries.

### 3.5. Lack of Sleep and Fatigue

Yet another source of fatigue shared by the participants is the lack of sleep. For a number of participants living in dorms far away from the construction sites where they work translates into having to wake up early in the morning (often between 4:30 a.m. and 5:30 a.m.) to get in the queue for using the bathroom facilities. The sites of work are in many instances between half-hour to 40 min away from the dorms, depending on the traffic. At the end of the day, after completing work, workers often return to their dorms between 8 p.m. and 9:00 p.m. The participants share that the limited rest during the weekdays often results in fatigue at work, which once again increased the chance of injuries. This is what *Alam* had to say:

I am tired at work, throughout the week. It is just the amount of time it takes to get to work and then to go back to the dorm room. And then in the dorm, it is hard to go to bed early because there are so many people in the room, and everyone is trying to do their own thing. And in the morning, I have to wake up very early so I can use the bathroom.

The long lines to go to the bathroom and get ready in the morning emerge across the interviews. The participants note the ways in which their schedules accompanied by work pressures result in injuries. The notion of feeling tired because of the lack of adequate facilities in the dorm is also voiced by *Sojol*, “The tiredness at work is what causes an accident. When you don’t have good sleep the night before and wake up early to make in time for the truck, you are going to be very tired.” This notion of feeling fatigue because of the lack of sleep, and resulting in workplace injuries is also shared by *Rehan*, “I don’t get more than 6–7 h of sleep each day. After the work, also many times in the heat, I get very tired, and then not enough rest. What to do?”

### 3.6. Mistreatment, Incivility, and Workplace Risks

The narratives shared by the participants often voice uncivil communication directed at the workers, and these experiences of incivility then translate into greater likelihood of errors and injuries at work. For *Boshir*, “When the boss shouts at me, I make mistakes because I am so worried.” Participants share stories of being mistreated at work by the supervisor and this threatens their health by making them vulnerable to accidents at construction sites. *Koreem* shares, “The boss is always shouting. Shouting and calling names. I get nervous, trying to hurry, and that’s when I make mistakes on the job.” On a similar note, *Sajal* shares, “I try very hard to follow what the boss says, but sometimes I can’t understand and the boss gets angry.” Not understanding is a theme that is reiterated in a number of interviews, suggesting that the lack of understanding leads to errors on the job. *Nitai* points out, “I can’t understand what the supervisor says, and that makes him angry. Then I am feeling the pressure, and end up dropping something or stepping on something.”

In these narratives, participants shared the ways in which being mistreated at work makes them vulnerable to making errors. *Chitta* posits, “When someone is shouting and saying things to you, you become absent-minded because you are upset. That’s when mistakes happen because you are not fully concentrating on the work.” Salient across the participant narratives is the interplay of being mistreated and the risks of workplace injuries, mediated by expressions of feelings of being upset. This is voiced by *Rajib*, “I have injured my hand at work because the boss was shouting at me and asking me to hurry. I could not understand what the boss was saying and so was trying to carry the crates even faster, and twisted my wrist.” Not understanding the supervisor is a theme that emerges across the in-depth interviews, with participants noting the pressures on the work that are related to not understanding the supervisor. This point is made by *Biram*, “I don’t understand what the boss says because he speaks in a different language, something that is not really English. The more I don’t understand, the more he starts shouting. And that makes me anxious, leading to mistakes I make.”

*Reyaz* shares, “The work is very hard, and I try to work hard so the boss will not abuse me. One time, I fell down while trying to hurry. The boss did not even let me rest properly, and I went on with the work.” This notion of “trying to hurry” is voiced throughout the interviews, with participants noting that their work is constituted amid demands of workplace efficiency and productivity. Similarly, articulates *Abdul*, “I am very tired with the work. Push my body really hard and the boss is still not happy. The boss will yell, and call us lazy. And I work hard so the boss will not call me lazy.” Errors then are made while “trying to hurry” in “finishing the job.” Worth noting here is the context of middle or lower middle class families in Bangladesh from which the workers often come, placing emphasis on civility of workplace interactions. Moreover, a number of the participants shared that they were educated until college, and were not used to the abusive behaviours of supervisors.

### 3.7. Health and Uncertainty

Sharing the uncertainties of their jobs, participants suggest that these uncertainties pose risks at work. Noting that the worry about finances is often distracting while on the job, they articulate the ways in which such distractions can cause workplace injuries. *Kabir* voices the following, “I am worried what will happen. How will I pay back all this money? Even at work, I am thinking about this. Sometimes this will take my mind away from work, and that can lead to an accident.” This point is also noted by Suman, who notes that “not knowing that I can keep this job, worrying about my family, worrying about the money in debt, I can’t focus on what I am doing. One time, I twisted my hand because I was not holding the wrench in the right way. I was worrying.” The anxieties related to income and the debt that most Bangladeshi workers have taken to come to Singapore are reiterated throughout the interviews.

The worries around high debts which are often taken to find the job in Singapore translate into mental pressure experienced by workers, which in turn, is articulated as a catalyst for workplace injuries. This is shared by Bashir, “When I worry, and have this pressure on my head, how can I concentrate on the job?” For *Dilbur*, “I paid the agent fees to get this job in Singapore. How can we afford the agent fees? My family is poor. So my family put on mortgage the only thing we have, the land. Now every month, I send money home. If there is delay in payment, then the interest goes up. So if salary does not arrive on time, I worry a lot.” The monthly income earned by construction workers such as *Dilbur* goes toward repaying the debt incurred. Delays in salary therefore result in greater levels of stress experienced by Bangladeshi construction workers in Singapore. This translates into being under constant mental pressure to return the money, articulated by the participants as the uncertainties of everyday life of migrant construction work.

The worries about debt are intertwined with worries about work, constituted around the uncertainty of work. Participants point toward the absence of adequate labor protections which leave a great deal of power in the hands of employers. “I am worried about my job, what is going to happen. Is the boss going to send back home? The boss can decide what to do. So I am worried about the job. I have the debt and can’t lose the job.” The uncertainties of the job translate into power in the hands of employers, with workers often experiencing this power as a source of everyday worries. For *Jamil*, “The job is not guaranteed. Now for example, a lot of workers are not working because the number of projects are less. So the company is going to cut people. I am thinking about that.” The sense of worry that *Jamil* experiences is often voiced by participants, sharing the challenges to health, safety, and wellbeing that are embodied in the uncertainties of work in the construction industries in Singapore.

## 4. Discussion

The narratives shared by the participants note the structural contexts of migrant construction work, situating the stories of workplace risks of injuries amid the broader ecology of migrant construction work. In response to the framework of workplace safety-based health communication interventions that emphasize individual behavior change [5,6], the narratives shared by the participants point toward the ways in which lived experiences with workplace safety are situated amidst the shifting contexts of work, attending to the overarching sociocultural contexts of workplaces as well as spaces of everyday living. Central to the findings of this study is the theorizing of risks of workplace injuries in the realms of spaces of everyday living, connecting workplace injuries to experiences with food, sleep, access to bathroom facilities, and transportation. In other words, workplace injuries are situated within the overarching structures of migrant construction work, attending to aspects of life beyond the work itself. Work, safety, and risks are tied to a plethora of contextual features of lives of migrant construction workers in Singapore, suggesting the interpenetration of contexts. One of the key theoretical contributions of this article therefore is in locating workplace injury in relationship to features of the broader environment of living experienced by migrant construction workers. These findings suggest that current safety strategies focusing on training (aimed at behavior change) may not be effective as long as workers are facing physical, financial, and emotional insecurity, constituted in the structures of the construction industry. Rather, a focus on behaviors needs to be complimented with policies ensuring access to quality food and shelter, organizational practices emphasizing good communication, and a broader culture of respect for migrant construction workers. The articulations of the workers point toward the necessity of a rights-based approach to workplace safety.

Moreover, the voices of the participants note the interplay between articulations of workplace injuries as embedded in the practices of day-to-day work, and their constructions of not really thinking about the risks of injuries at work. The participants share that because they witness and experience workplace injuries, they think of these injuries as parts of their everyday work. Their lived experiences with risks to health and witnessing these risks to health also shape the ways in which the participants situate the workplace risks to health as being beyond their everyday cognitions. Their vulnerabilities to risks of workplace injuries shape the ways in which the migrant construction workers participating in this project come to make sense of their work; in this context, “not thinking about” the risks at work becomes a way for the participants to continue working amid their everyday vulnerabilities to these risks. Therefore, to avoid thought about workplace injuries is a coping mechanism in response to a sense of extreme vulnerability and everyday susceptibility to workplace injuries. A culturally situated narrative of destiny draws upon the sense of inevitability that is tied to the structures of construction work. The culture-structure-agency framework of the CCA offers a lens for interpreting the ways in which cultural stories about fate emerge in enactments of agency (not thinking about risks) and are shaped by structures (risks in construction work).

Also, the participant narratives attend to the role of power in the realm of workplace injuries and health seeking [4,15,16,28]. For instance, the power of the employer in controlling health care resources shapes the experiences of participants with seeking out healthcare after the injury, access medical leave, and secure compensation. Participant narratives noting that the health care providers for treating the injuries are assigned by the companies point to the ways in which the prescription of the medical certificate is constituted amidst power. In these narratives, the doctor treating the injury is aligned with the interests of the company, depicting the ways in which the power in overarching organizing structures of work is tied to the health seeking options available to migrant construction workers in the backdrop of workplace injury. Note here the overarching role of structures in shaping the access to healthcare after workplace injuries [15,24,25,27]. The worries about being economically viable after an injury depict the vulnerabilities of migrant construction work, and also point to the precarious nature of such forms of work.

In voicing the causes of workplace injuries, the Bangladeshi construction workers participating in this project point to the organizing structures that constitute their everyday lives. The structures extend beyond the spaces of work in the construction industry to spaces of everyday life, depicting the features of structures of living that are closely intertwined. Not having access to healthy, safe, and clean food for instance can result in threats to health of migrant construction workers. Workers share the ways in which not having adequate and proper food results in fatigue, which in turn increases the likelihood of injuries in the workplace. The experience with food insecurity is intertwined with experiences of risks at the workplace. The emergence of food as a key element in the discursive space is tied to the centrality of food in Bangladeshi life. On a similar note, the lack of adequate sleep results in fatigue, which in turn results in risks of workplace injury. The lack of sleep in turn is constituted in relationship to the distance of workplaces from spaces of living, transportation, lack of adequate bathroom facilities, and the shared nature of living spaces where many workers sleep in one room. The articulation of risks of workplace injury within the broader ecology of life of migrant construction workers points toward an integrated framework of workplace injuries in construction work that foregrounds arrangements of living that are accessible to construction workers. Articulations of workplace safety cultures are complemented by structural features of living such as food, shelter, and transportation.

Moreover, the participant narratives point to the ways in which workplace productivity and efficiency targets place workers in the construction industry at risk of injury. Noting that the drive to efficiency translates into being rushed on the job, workers share the challenges they experience when rushed on the job, increasing the likelihood of making errors. The anchoring of construction work in narrow productivity targets increases the risks of injury at work. These culture-centered articulations thus call for shifting the overarching narratives of productivity and efficiency that emerge as monolithic narratives driving the nature of work. In the voices of the participants, the overarching framework of productivity as the driving force for work translates into affective experiences of anxiety, which in turn, result in likelihood of errors.

Finally, the participants narrate the role of communication shaping their experiences of work, the risks they experience at work, and the anxieties that are intertwined with these risks. In the voices of the workers, the threats to dignity reflected in experienced “being shouted at” result in experiences of anxiety, which in turn result in errors and corresponding injuries. For a number of participants, language and communication barriers are key challenges that exacerbate the experiences of incivility. Although a number of participants are conversant in English (with a substantive number having a college education), the different accents and linguistic styles used by the supervisor result in miscommunication and/or lack of understanding. In a wide range of narratives, participants share the ways in which they are “shouted at” for not understanding the instructions given by the supervisor. These experiences suggest the importance of developing adequate communication programs for supervisors in construction sites that address issues of incivility, impolite communication, and mistreatment. Moreover, these experiences suggest development of appropriate organizational policies for building cultures of respect for workers. In addition, appropriate policy solutions are needed that offer exit options to workers, addressing the unequal power relations within which they toil.

Challenging a behaviourally driven framework that is focused on developing effective health/risk messages, the voicing of overarching structures of work and everyday living attends to the context of migrant construction work. Contexts shape the ways in which work is experienced, carried out, and negotiated. Moreover, contexts shape the ways in which workplace errors and injuries are understood and experienced. Structures constitute these contexts, working hand-in-hand with cultural narratives and interpretive frameworks that are anchored in culture [3,5,9,17,19,23,30]. Also worth noting are the linkages between the immediate contexts of workplace risks and the broader contexts of familial debt and economic responsibility to family spatially located in Bangladesh. The participants in our culture-centered project contextualize the vulnerabilities of health that flow from Bangladesh to Singapore, and point to the neoliberal global policies that produce precarious conditions that threaten human health and wellbeing.

Adding to a growing body of literature on immigration and health communication grounded in the tenets of the CCA [15,19,24,27,28], this article highlights the role of work as a defining feature of migrant health amid neoliberal flows of labour. Health is constituted in the vulnerabilities of/at work, connected to features of everyday living. Work and life in this sense are porous, flowing from each other, and interconnected in a broader framework of marginalization amid organizing structures of construction work. The precarious nature of construction work with limited to no security on the job renders workers vulnerable, depicting the ways in which power constrains and enables the nature of work, and the constructions of health at work sites. The uncertainties of work, accompanied by the economic uncertainties experienced by the construction workers renders them vulnerable, producing anxieties, difficulties in concentrating on the job, and risks of workplace injuries. The health of migrant construction workers is threatened by the often unprotected working conditions they experience. Particularly salient in this context is the development and implementation of adequate policy frameworks and comprehensive interventions that offer the needed protections to migrant construction workers. Moreover, when workers have access to spaces where they can represent their voices, they can participate in shaping the living and working conditions of their everyday lives. The urban subaltern in the neoliberal city, voices pathways of representation and recognition by challenging structures that constrain subaltern agency, and by interrogating the overarching organizing logics of structures. Future scholarship on immigrant health ought to examine the challenges of communication work that seeks to interrogate the structures and make entry points for listening to subaltern voices, both in worksites as well as in policy spaces that govern work and life of migrant construction workers. One of the limitations of this study is its focus on a small section of migrant construction workers. The in-depth interviews reported for this paper specifically draw from the experiences of Bangladeshi construction workers in Singapore. Future scholarship ought to examine the experiences with construction work among other communities of workers, specifically exploring the similarities and differences in patterns of experiences.

## 5. Conclusions

The CCA co-creates entry points for inverting the marginalization experienced by contractual migrant workers in the construction industry, and it does so by disrupting the dominant discourses of migrant construction work. For instance, dominant articulations of migrant labour to be strategically managed through efficiency, awareness, and effective management of workplaces [25,26,27,28] is shifted into the hands of migrant workers, who point toward the necessity for creating opportunities of articulation that offer contractual migrant workers modes of access to addressing the health threats they experience, and opportunities for meaningfully participating in platforms that enable their articulations to be heard in ways that matter [28,29,30]. The interplays of culture, structure, and agency depict the ways in which workers enact their agency by drawing on cultural stories, situated amid the structures of construction work. The narratives sharing the role of health service providers who work in collusion with employers to deny medical leave or treatment to workers points toward the need for the development of intervention measures that hold health service providers and employers accountable in addressing the health needs of construction workers. Similarly, the experiences of being mistreated by employers and the effects of such mistreatment on workplace health risks point toward the importance of addressing the stigmatization of work and the cultural codes of inequality that are built into workplace management practices. The articulations of health as dignity point toward the need for developing health communication that is directed at addressing the overarching inequalities in the distribution of power in construction industries in the global North that employ migrant workers, and the ways in which these inequalities play out in relationships between workers and supervisors in construction sites.
